# Poetry

**Published:** 1870-03

**Authors:** 


[WHILE the candidates for graduation, at the recent Commencement of Rush Medical College, were responding, in writing, to Prof. Gunn's interrogatories, a first-course student perpetrated and sent down the following impromptu :]

Oh! Dr. Gunn, it is such fun
To stand examination.
While sitting high, who else but I
Can give the explanation?

I.

Of abscess,* signs of which are pains
Above the sacral bone,
A weakened back, and general lack
Of health, and strength, and tone.
And when the pus shall make a fuss,
And down the psoas burrow,
Upon the thigh will appear high
Suspicions, like a furrow.
And if of wit you've not a bit —
For hernia should mistake it,
'Tis my belief you'll come to grief —
A cure you will not make it.
I think there is, — so Druitt says, —
A kind that does not burrow,
But keeps behind and is the kind
That makes the patient sorrow.
It gives him chills, and often fills
His nights with pain and woe,
With hectic blush his cheek will flush,
He thinks he soon must go.

II.

How shall we treat it? Why, thus we'll greet it:
On beef we'll feed him well.
And if at length he keeps his strength,
Prognosis good we'll tell.
And when it points, why use the lance
And let the matter out.
'Tis of no use to keep him close,
But make him ride about.

III.

Of number three, just this I'll say:
You'll find the scrotum full;
'Twill feel like worms had there their home,
And downward it will pull.
The venous blood, an ebbing flood,
Comes down the spermatic vein.
It will not flow where it should go,
But settles back again.
Just tie the vein, and then, I ween,
No more will you be wanted;
The weight above you will remove,
Your service will be vaunted.

* Psoas.

IV.

Of cancer? Well, I cannot tell
Just why it should appear;
But this I know, 'twill faster grow
The more we interfere;
Unless to save, with courage brave,
We use the surgeon's knife:
I've heard you say, "perchance it may
Prolong the patient's life."
I think you said "'twas called colloid,"
The alveolar kind.
Where it loves best to make its nest
I cannot call to mind.
Its appearance gross, I must confess,
Like jelly somewnat looks —
Of watery hue, transparent, too,
And full of little nooks.
Peculiar cells its substance fills,
The microscope will show.
But what they are, I am not sure —
*You* surely ought to know.

V.

A broken knee? Oh, yes, I see
The fracture is transverse;
'Twill lengthwise go, and radiate, too,
Which makes the matter worse.
The truth to tell, the knee will swell —
And may I be so bold? —
The lazy wretch his leg can't stretch,
The "rectus" has lost his hold.
Now, a roller find and tightly bind
The thigh from ilium down,
And then, at last, just stick it fast,
If "adhesive" can be found.
The leg keep straight, and keep it late,
Upon a splint extend;
Six weeks, you know, before 'twill do
To let it freely bend.

Why, bless me! so I've got through,
And it is getting late, —
This more I'll tell — I wish quite well
I were a graduate.

A First-Course Student.

